# Does the separating of hospital revenue from drug sales reduce the burden on patients? Evidence from China

**DOI:** 10.1186/s12939-020-01363-5

**Published:** 2021-01-06

**Authors:** Lele Li, Qiao Yu

**Affiliations:** School of Public Policy and Management, Tsinghua University, Haidian District, Beijing, 100084 China

**Keywords:** Separating of hospital revenue from drug sales (SHRDS), Healthcare insurance reimbursement (HIR), Healthcare expenditure, Difference in difference model (DID)

## Abstract

**Background:**

Since 2015, all pilot cities of public hospital reform in China have allowed the zero-markup drug policy and implemented the policy of Separating of Hospital Revenue from Drug Sales (SHRDS). The objective of this study is to evaluate whether SHRDS policy reduces the burden on patients, and to identify the mechanism through which SHRDS policy affects healthcare expenditure.

**Methods:**

In this study, we use large sample data of urban employee’s healthcare insurance in Chengdu, and adopt the difference in difference model (DID) to estimate the impact of the SHRDS policy on total healthcare expenditures and drug expenditure of patients, and to provide empirical evidence for deepening medical and health system reform in China.

**Results:**

After the SHRDS policy’s implementation, the total healthcare expenditure kept growing, but the growth rate slowed down between 2014 to 2015. The total healthcare expenditure of patients decreased by only 0.6%, the actual reimbursement expenditure of patients decreased by 4.1%, the reimbursement ratio decreased by 2.6%. and the drugs expenditure dropped by 14.4%. However, the examinations expenditure increased by 18.2%, material expenditure increased significantly by 38.5%, and nursing expenditure increased by 12.7%.

**Conclusions:**

After implementing the SHRDS policy, the significant reduction in drug expenditure led to more physicians inducing patients’ healthcare service needs, and the increased social healthcare burden was partially transferred to the patients’ personal economic burden through the decline in the reimbursement ratio. The SHRDS policy is not an effective way to control healthcare expenditure.

## Introduction

**Fig. 1:**
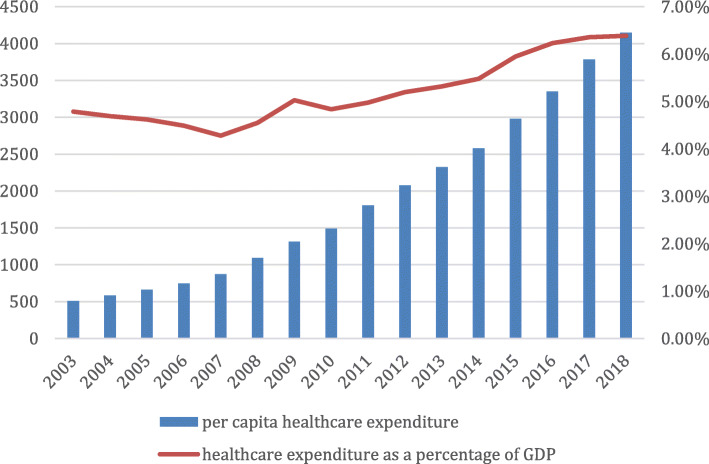
The per capita healthcare expenditure and healthcare expenditure as a share of GDP in China from 2003 to 2018. Note: The unit of healthcare expenditure is Yuan (CNY), and the healthcare expenditure as a percentage of GDP is %. Data sources: Annual statistical yearbook of China

As the economy developed, consumption behavior have undergone great changes. The demand for healthcare is increasing, and the soaring healthcare expenditure has become a global concern for both developing and developed countries [1]. This burden is worsened by fast-growing aging population. From Fig. 1, we can find the per capita healthcare expernditure and healthcare expenditure as a share of GDP in China fom 2003 to 2008. Compared to most developed countries, China faces even greater challenges in controlling its growth in healthcare expenditure [2]. In terms of the composition of total healthcare expenditure, drug expenditure is the highest in China. In 2014, drug expenditure accounted for 48.3 and 38.3% of the total outpatient and inpatient healthcare expenditure respectively. Both forms of expenditures are much greater than the average level of 17% in OECD countries [3]. Hospital revenue from drug sales, which is under supply-induced demand, is the fundamental reason for the high proportion of drug expenditure in China [4, 5].

Drug-sales-led hospital revenue has been a phenomenon of the Chinese healthcare system since the 1950s. The phenomenon is a result of the high added value of medicines by physician labor, the economic benefits of hospitals with high profits of medicines, and the normal operation of hospitals. Prior to the new round of health care reform in 2009, public medical sectors were allowed to impose a 15% profit margin for drugs sales to compensate for operation costs. Without drug sales, receipts for public hospitals in China mainly comes from medical service charges and government subsidies. However, due to the limited financial support from the government and the price ceiling imposed on healthcare service fees, the main income of the hospital comes from a 15% profit margin for drugs sales, which is also one of the main reasons for the high prices of drugs and seeing a doctor. Therefore, in order to solve the problem of health care’s limited access and prohibitive costs (commonly known as *kan-bing-nan, kan-bing-gui* in Chinese), the central government officially launched a new round of medical and health system reform in 2009, with the SHRDS policy at the core of the reform, hoping to disentangle hospital revenue from drug sales, to contain the rapid growth of healthcare expenditure, as well as reduce the economic burden, and meet the healthcare needs of the people.

The SHRDS policy has been around for over a decade in China, but few researchers have evaluated its impact, especially for economic burden on patients. In this study, we address three research questions: (1) what is the effect of the SHRDS policy on healthcare expenditure? (2) Does the SHRDS policy really reduce the burden on patients? (3) What is the mechanism through which the SHRDS policy affects healthcare expenditure? We believe that the process of medical and health system reform is a complicated social system engineering, although the increase of public medical and health input plays an important role, the improvement of reducing healthcare expenditure, as well as the economic burden on patients are much more vital. Therefore, it is necessary to evaluate whether the SHRDS policy actually reduce the burden on patients, identify the mechanism of effect of SHRDS policy on healthcare expenditure, and provide empirical evidence for deepening the medical and health system reform in China.

## Methods

### Institutional background

In order to implement the SHRDS policy in public hospitals, the government of Chengdu clarified the scope and time of the SHRDS policy, and requested all public hospitals at the county-level to abide by the SHRDS policy. From October 1, 2013, a 15% profit margin for drug sales (excluding Chinese medicine drinks) would be canceled, and the mechanism of hospital revenue from drug sales would be eliminated to reduce the burden of healthcare expenditure for patients. In 2014, the government of Chengdu clearly required 33 county-level public hospitals to cancel a 15% profit margin for drug sales. The implementation of the SHRDS policy in Chengdu county-level public hospitals entered a new stage from October 1, 2014. Because the county-level public hospitals in the urban area are very similar. The situation of medical conditions, technical level and medical ability between the SHRDS and the Non-SHRDS county-level public hospitals is relatively consistent, which provides good conditions to use the DID method for evaluating the effectiveness of the SHRDS policy.

According to the SHRDS policy in Chengdu, the elimination of a 15% profit margin for drug sales and the establishment of a multi-channel compensation mechanism are the core elements of the SHRDS policy in Chengdu. Increasing financial subsidies and adjusting the price of healthcare services are the main means of multi-channel compensation mechanisms. In order to compensate for the loss in potential drug revenue, the government simultaneously raised fees for medical services that, for historical reasons, were previously set far below actual costs [6]. Generally speaking, after the SHRDS policy, the price of medicine will decrease due to the cancellation of a 15% profit margin for drug sales, and the healthcare expenditure of the patient will also decrease, thereby reducing the total healthcare expenditures for the patient. However, under the existing compensation mechanism, if the increase in the price of financial subsidies and healthcare services is not enough to make up for the loss of profits from the decline a 15% profit margin for drug sales, hospitals may use information advantages to induce patients to increase examinations and nursing among other aspects in order to maintain profit levels. The demand for healthcare services increases the expenditure of patients on other healthcare services. At this time, the total healthcare expenditure of patients may not necessarily decrease, and may even increase. Therefore, after the implementation of the SHRDS policy, whether the total healthcare expenditure for patients is rising or falling is theoretically uncertain, and further data analysis is needed.

### Data source and descriptive statistics

The data used in this paper is from the very large sample data of urban employee’s HIR provided by Chengdu healthcare insurance administration. The data mainly includes the healthcare record home page information (disease diagnosis, ICD-10 code, outcome, name and grade of hospitals, etc.), healthcare expenditure reimbursement information (actual reimbursement expenditure, reimbursement ratio, total healthcare expenditure, drug expenditure, examination expenditure, material expenditure, nursing expenditure, bed expenditure, blood expenditure, surgery expenditure, etc.) and the patients’ basic characteristics (age, gender, type of diseases, length of hospital stay, etc.).This study takes October 1, 2014 as the center point for the second batch of county-level public hospitals to SHRDS in Chengdu. The time range for selecting data is from January 1, 2013 to December 31, 2015. We choose the Non-SHRDS county-level public hospitals as the control group and SHRDS county-level public hospitals as the experiment group. All expenditure variables in the study are converted to 2014 yuan using the Consumer Price Index (CPI). It is better to reflect the changes before and after SHRDS of county-level public hospitals in Chengdu and to control patients’ individual characteristics, which makes the analysis more rigorous and the conclusion more reliable.

**Fig. 2:**
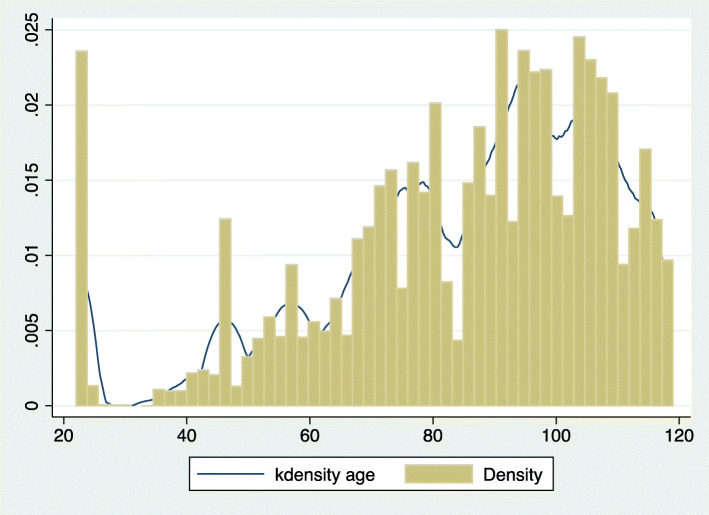
The kdensity and density of age

The data sample size contains 514,631 HIR records (Table 1), including 121,130 observations in 2013, 160,482 observations in 2014 and 233,019 observations in 2015. Of the observations, 52% were male and most of them were elderly patients with an average age of 86.25 years old. This finding is corroborated in Fig. 2. From Table 2, we can also find the percentiles of the age distribution with 10%, 25%, 75% and 90%. The majority of the patients received medical treatment in Grade II Level A hospitals, and the disease types were mainly common diseases. Most of the patients recovered after receiving medical treatment in the hospital.

**Table 1:** Basic characteristics of sample observations

Variable name	Full sample (*n* = 514,631)		In 2013 (*n* = 121,130)		In 2014 (*n* = 160,482)		In 2015 (*n* = 233,019)	
	Mean	Std.Dev.	Mean	Std.Dev.	Mean	Std.Dev.	Mean	Std.Dev.
Gender	0.52	0.50	0.53	0.49	0.52	0.50	0.52	0.50
Age (years)	86.25	23.81	86.37	24.35	86.34	23.56	86.10	23.68
Average length of hospital stay (days)	9.42	7.73	9.94	8.86	9.42	8.00	9.15	6.85
^a^Classification of hospitals	1.79	0.49	1.61	0.67	1.78	0.51	1.90	0.30
^b^Diseases types	1.85	0.47	1.83	0.49	1.84	0.49	1.87	0.44
^c^Outcome	1.99	0.36	2.00	0.39	1.99	0.39	1.98	0.31

Gender: Male = 0, Female = 1. Classification of hospitals: Grade II Level C = 0, Grade II Level B = 1, Grade II Level A = 2.Diseases types: Chronic diseases = 0, Critical diseases = 1, Common diseases = 2. Outcome: Death = 0, Transfer = 1, Rehabilitation =2, Others = 3

^a^Classification of hospitals are divided into Grade III hospitals, Grade II hospitals, Grade I hospitals according to their functions and roles. Grade I hospitals include community health centers and township health centers that directly provide prevention, medical care and rehabilitation services to residents. Grade II hospitals are secondary hospitals that provide comprehensive medical services to a region, undertake some teaching and scientific research tasks. Grade III hospitals are tertiary hospitals that provide high-level specialized medical services and undertake advanced teaching and scientific research tasks. In this study, the percentage of Grade II Level C, Grade II Level B, and Grade II Level A are 83%, 12%, and 5%, respectively

^b^Chronic disease refers to diseases that do not constitute infection and have long-term accumulation of disease form damage (i.e. high blood pressure, diabetes, etc.). Critical diseases refer to diseases that are costly to treat and severely affect the normal work and life of patients and their families for a long period of time (i.e. cancer etc.). In this study, the percentage of Chronic disease, Critical diseases, and Common diseases are 5%, 6%, and 89%, respectively

^c^Transfer refers to patients that move from one hospital to another hospital. Rehabilitation refers to the patients that are cured and leave the hospital. Others refers to the outcomes of patients except death, transfer and rehabilitation. In this study, the Transfer, Rehabilitation, and Others are 3%, 91%, and 6%, respectively

**Table 2:** The percentiles of the age distribution

Percentiles	10%	25%	75%	90%
Age	53	73	104	113

We used Stata 14.0 for all analyses in this paper. The statistical description results of variables are shown in Table 3. The total healthcare expenditure of patients was 5165.50 Yuan (CNY)(USD $737.9) in 2013, 5639.81 Yuan(CNY) (USD $805.6)in 2014 and 5914.81 Yuan(CNY)(USD $844.9) in 2015, respectively. The total healthcare expenditure kept increasing, but the growth rate slowed down from 2014 to 2015.The drug expenditure of patients were 2224.51 Yuan(CNY)(USD $317.7) in 2013, 2154.91 Yuan (CNY)(USD $307.8)in 2014 and 2017.82 Yuan (CNY)(USD $288.2)in 2015,which accounted for 43.06, 38.21 and 34.11% of the total healthcare expenditure, respectively. The drug expenditure of patients and its proportion in the total healthcare expenditure was declining. However, the expenditure of examinations and materials for patients increased significantly from 2013 to 2015, especially in SHRDS hospitals that implemented the SHRDS policy.

**Table 3:** Descriptive statistics of the main variables

Variable name	Full sample		SHRDS hospitals		Non-SHRDS hospitals	
	Mean	Std.Dev.	Mean	Std.Dev.	Mean	Std.Dev.
A: In 2013						
Total healthcare expenditure	5165.50	5713.70	5669.82	6254.44	3839.95	3634.74
Drug expenditure	2224.51	2688.40	2436.06	2950.43	1668.49	1708.03
Examination expenditure	1230.62	1321.01	1419.08	1430.05	735.29	786.07
Material expenditure	370.54	1511.02	413.99	1652.62	256.35	1042.95
Nursing expenditure	140.55	311.15	140.56	340.41	140.53	216.10
Bed expenditure	245.11	273.38	249.21	289.31	234.31	225.90
Blood expenditure	32.75	293.91	41.43	335.10	9.93	132.56
Surgery expenditure	204.13	586.29	220.15	620.77	162.01	481.57
Reimbursement ratio (%)	69.38	0.17	70.03	0.17	67.65	0.16
Deductible line	259.34	99.57	278.24	95.79	209.67	91.92
Actual reimbursement expenditure	3861.68	4533.19	4261.11	4989.29	2811.82	2759.18
B: In 2014						
Total healthcare expenditure	5639.81	6188.36	6030.62	6499.67	3805.52	3959.32
Drug expenditure	2154.91	2735.83	2314.13	2888.58	1407.61	1665.87
Examination expenditure	1494.39	1529.00	1624.83	1594.05	882.18	964.24
Material expenditure	501.65	1787.69	518.08	1874.33	424.55	1303.64
Nursing expenditure	175.09	252.29	182.08	265.58	142.28	173.33
Bed expenditure	226.63	233.72	234.53	245.93	189.59	159.59
Blood expenditure	32.83	306.89	37.44	329.44	11.21	162.17
Surgery expenditure	234.44	640.03	239.46	655.69	210.87	560.15
Reimbursement ratio (%)	69.92	0.16	71.51	0.16	62.43	0.15
Deductible line	268.70	93.59	274.52	94.41	241.42	84.44
Actual reimbursement expenditure	4212.72	4850.74	4562.13	5103.83	2572.81	2905.62
C: In 2015						
Total healthcare expenditures	5914.81	6380.34	6132.23	6541.76	3850.99	4025.03
Drug expenditures	2017.82	2571.89	2106.76	2650.19	1173.57	1394.98
Examination expenditure	1790.27	1853.52	1870.22	1906.39	1031.39	954.99
Material expenditure	532.32	1877.55	537.36	1916.37	484.50	1457.71
Nursing expenditure	176.92	239.73	178.30	242.21	163.78	214.32
Bed expenditure	211.40	216.52	215.12	221.54	176.16	156.97
Blood expenditure	32.84	295.46	34.87	306.19	13.54	159.97
Surgery expenditure	243.05	654.83	245.23	664.19	222.28	556.42
Reimbursement ratio (%)	70.87	0.16	71.69	0.16	63.05	0.14
Deductible line	269.42	96.11	272.42	96.79	240.88	84.17
Actual reimbursement expenditure	4452.12	5055.21	4650.66	5197.99	2567.60	2781.08

The unit of all expenditure variables is Yuan (CNY), and the unit of reimbursement is %. All expenditure variables in the study are converted to 2014 price level using the Consumer Price Index (CPI)

**Fig. 3:**
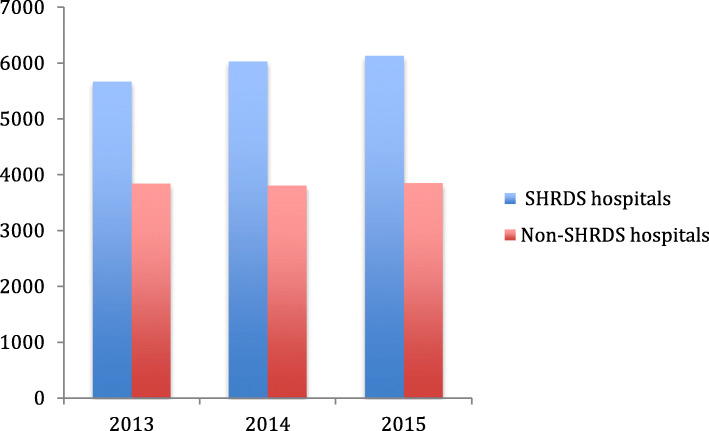
Total healthcare expenditure for patients in SHRDS hospitals and Non- SHRDS hospitals. Notes: The Y-axis shows the average total healthcare expenditure of the patients (unit: Yuan[CNY]) and X-axis shows the year

**Fig. 4:**
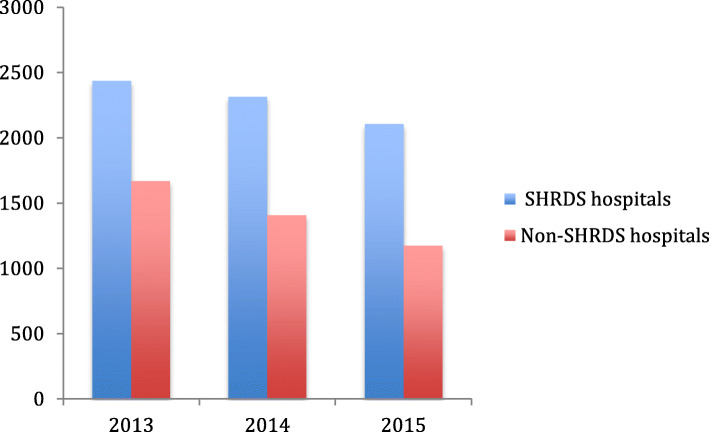
The drug expenditure for patients in SHRDS hospitals and Non- SHRDS hospitals. Notes: The Y-axis shows the average drug expenditure of the patients (unit: Yuan[CNY]) and X-axis shows the year

**Fig. 5:**
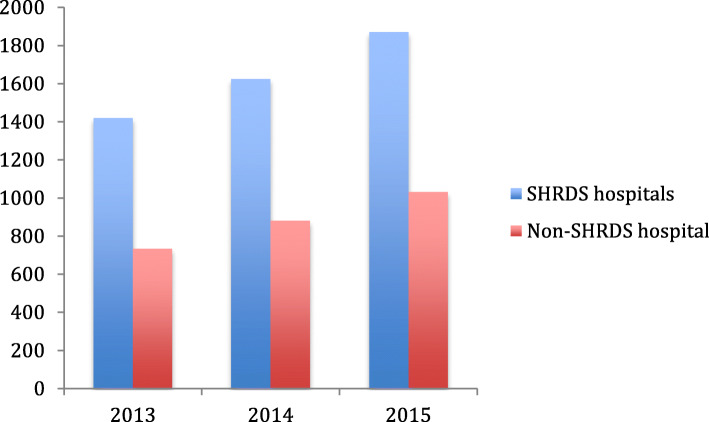
The examination expenditure for patients in SHRDS hospitals and Non- SHRDS hospitals. Notes: The Y-axis shows the average examination expenditure of the patients (unit: Yuan [CNY]) and X-axis shows the year

**Fig. 6:**
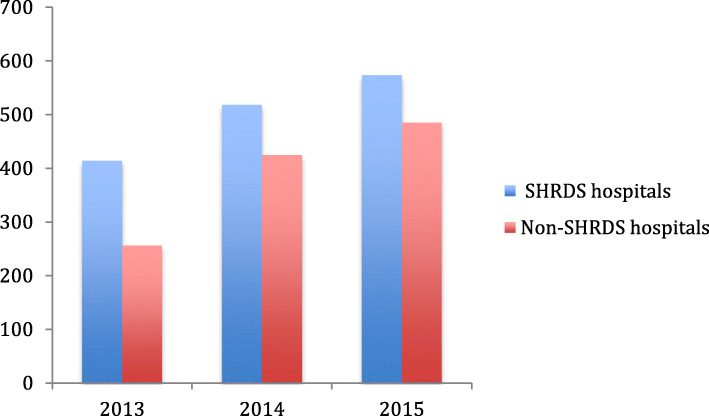
The material expenditures for patients in SHRDS hospitals and Non- SHRDS hospitals. Notes: The Y-axis shows the average material expenditures of the patients (unit: Yuan [CNY]) and X-axis shows the year

From Figs. 3, 4, 5 and 6, we can find the differences in the performance of various healthcare expenditure for patients in SHRDS hospitals and Non-SHRDS hospitals. From Fig. 3, we find that the total healthcare expenditure of SHRDS hospitals increased significantly in 2013 to 2015, while the total healthcare expenditure of Non-SHRDS hospitals were relatively stable during the same period. From Fig. 4, we find that the drug expenditure of patients in SHRDS hospitals and Non-SHRDS hospitals declined significantly in 2013–215, but the expenditure of drugs in SHRDS hospitals declined more significantly. From Fig. 5 and Fig. 6, we find that the expenditure of examination and materials for patients in SHRDS hospitals increased significantly, but the increase in the expenditure of examinations and materials for SHRDS hospitals was higher than that of Non-SHRDS hospitals.

### Model setting

#### Model for estimating the SHRDS policy

We used DID method to estimate the impact of the SHRDS policy on the total healthcare expenditure and the structure of healthcare expenditure. In many economic studies at home and abroad, the DID method is widely used for policy effect evaluation [7–10]. Following the Chen et al. [10], we use the following model to estimate the impacts of the SHRDS policy formally.

1 $$ {Y}_{ikt}={\alpha}_0+{\alpha}_1{Policy}_{ikt}+{\alpha}_2X+{\theta}_k+{\mu}_t+{\epsilon}_{ikt} $$

The dependent variable *Y*_*ikt*_*Y*_*ikt*_ indicates a series of expenditure variables(actual reimbursement expenditure, reimbursement ratio, total healthcare expenditures, drug expenditure, examinations expenditure, material expenditure, nursing expenditure, etc.)with patient *i*, in hospital *k*,and the time of year *t.*All the dependent variables in this study, except for reimbursement ratio, are estimated in logs because the distribution of healthcare expenditure is typically skewed *Policy*_*ikt*_ indicates that if the SHRDS policy has been implemented with patient *i*, in hospital *k*,and the time of year *tt*,it equals one(1).Otherwise, it equals zero(0). *XX* represents a series of control variables, including gender, age, outcome, type of diseases, and so on. *θ*_*k*_ represents hospital fixed effects and *μ*_*t*_ represents time fixed effects.

The effectiveness of the DID method depends on whether the change in the outcome variables of the experimental and control groups before and after the policy is independent of the control group. That is to say, in the absence of policy change, the outcome variables of the experimental and control groups did not have systematic or statistically significant differences before and after the policy occurrence. Since we can’t observe the counterfactual situation in the absence of policy change, we can’t directly test the DID recognition hypothesis. However, we can indirectly test the DID recognition hypothesis by examining whether the outcome variables of the experimental group and the control group have the same time trend before the policy occurs. That is the pre-policy pretend test. This is also a common practice in empirical literature using the DID method.

#### Model for pre-policy pretend test

Before conducting an empirical analysis, we first make the pre-policy pretend test. According to the SHRDS policy in Chengdu, we selected the time of sample before October 12,014 when there are Non-SHRDS hospitals. So we used the DID model to test whether there were significant changes in the total healthcare expenditure and the structures of healthcare expenditure in county-level public hospitals around January 1, 2014.In the case of the establishment of the DID identification hypothesis, because there was no impact of policy changes around January 1, 2014, the changes in the total healthcare expenditure and the structures of healthcare expenditure in county-level public hospitals before and after January 1, 2014 were publicized. There should be no statistically significant differences between public hospitals. Specifically, we use a model setting similar to eq. (1).

2 $$ {Y}_{ikt}={\beta}_0+{\beta}_1{PesudoPolicy}_{ikt}+{\beta}_2X+{\theta}_k+{\mu}_t+{\varepsilon}_{ikt} $$

The difference between eq. (1) and eq. (2) is that if the SHRDS policy has been implemented with patient *i*, in hospital *k* and the time of year *t* after January 1, 2014, *PesudoPolicy*_*ikt*_*PesudoPolicy*_*ikt*_ equals one(1).Otherwise, it equals zero(0).It is assumed that the Chengdu County-level public hospitals have implemented the SHRDS policy on January 12,014. Obviously, this is a virtual policy changes shock. If there is no statistically significant difference in the *β*_1_, there is no statistically significant difference in the changes in the total healthcare expenditures and the structures of healthcare expenditure before and after January 12,014.

## Results

### Empirical results of the results of pre-policy pretend test

As seen from Tables 4 and 5, they showed the test results of the DID recognition hypothesis. In terms of total healthcare expenditure and reimbursement expenditure, we found that the total healthcare, nominal reimbursement, actual reimbursement expenditure and reimbursement ratio increased by 3.8% (e^0.038^–1, *p*-value = 0.452), 5.5% (e^0.054^–1, *p*-value = 0.375), 14.4% (e^0.135^–1, *p*-value = 0.557), and 6.2% (e^0.060^–1, *p*-value = 0.187), respectively. In terms of structures of healthcare expenditure, we found that drug and nursing expenditure increased by 8.3% (e^0.080^–1, *p*-value = 0.439) and 13.8% (e^0.130^–1, *p*-value = 0.620), and inspection and material expenditure decreased by 15.1% (e^− 0.164^-1, *p*-value = 0.385) and by 31.4% (e^− 0.377^-1, *p*-value = 0.517).

**Table 4:** Test of DID identification hypothesis: total healthcare expenditure and reimbursement expenditure

Variables	Total healthcare expenditure	Nominal reimbursement expenditure	Actual reimbursement expenditure	Reimbursement ratio
*PesudoPolicy*	0.038 (0.007)	0.054 (0.007)	0.135 (0.008)	0.060 (0.001)
Covariates variables	Y	Y	Y	Y
Time fixed effects	Y	Y	Y	Y
Hospital fixed effects	Y	Y	Y	Y
Observations	281,612	281,612	281,612	281,612
R-squared	0.23	0.26	0.28	0.28

Covariates variables include the gender, age, outcome, type of diseases, and so on. Robust standard errors are reported in parentheses

**Table 5:** Test of DID Identification Hypothesis: structures of healthcare expenditure

Variables	Drug expenditure	Inspection expenditure	Material expenditure	Nursing expenditure
*PesudoPolicy*	0.080 (0.009)	-0.164 (0.009)	-0.377 (0.015)	0.130 (0.007)
Covariates variables	Y	Y	Y	Y
Time fixed effects	Y	Y	Y	Y
Hospital fixed effects	Y	Y	Y	Y
Observations	281,612	281,612	281,612	281,612
R-squared	0.18	0.29	0.06	0.09

Covariates variables include the gender, age, outcome, type of diseases, and so on. Robust standard errors are reported in parentheses

However, they were all not statistically significant (at the 10% level). That is to say it satisfies the pre-policy counterfactual. We did not find a statistically significant difference in the total healthcare expenditures and the structures of healthcare expenditure in public hospitals around January 12,014, which supports our DID recognition hypothesis.

### Empirical results of total healthcare expenditure and reimbursement expenditure

In 2009, a new round of medical and health system reform was officially launched, and the SHRDS policy was taken as the core content of the reform. We can find the impact of the SHRDS policy on the total healthcare expenditure and the reimbursement expenditure for patients (Table 6). After the implementation of the policy of SHRDS, the total healthcare expenditure decreased by 0.6% (e^− 0.006^-1, *p*-value = 0.340), but it is not statistically significant (at the 10% level) and just a small decline. The SHRDS policy has a certain effect on reducing the economic burden of the people, but the effect on reducing the total healthcare expenditure for patients is very limited. To understand whether the SHRDS policy play a greater role may require a longer period of time for policy pilots. At the same time, after the policy of SHRDS, the actual reimbursement expenditure of patients decreased significantly by 4.1%(e^− 0.004^-1,*p*-value = 0.000), and the reimbursement rate decreased significantly by 2.6%(e^-0.026^-1,p-value = 0.000), which indicates that the SHRDS policy has a certain effect on reducing the total healthcare expenditure of patients, but it also reduces the reimbursement expenditure and increases the personal economic burden of the patients. Zhang et al. [11] found that the reform of county-level public hospitals significantly increased the healthcare expenditure of patients covered by the new rural healthcare insurance, and the self-pay expenditure of patients increased significantly by 15.4%, which is consistent with the findings of this study. However, why does the SHRDS policy reduce the total healthcare expenditure of patients, while it also increases the personal economics burden of patients? As the SHRDS policy removed the 15% markup for drug sales in public hospitals, the hospitals’ income dropped drastically. The government compensated the hospital with financial subsidies and price adjustment for health services. Obviously, it will increase the burden on the government’s public finances. So, the reimbursement expenditure will be reduced and the personal economic burden of patients will increase. So, the final increase in the public financial burden will be partially transferred to the personal economic burden of patients.

**Table 6:** Total healthcare expenditure and reimbursement expenditure

Variables	Total healthcare expenditure	Nominal reimbursement expenditure	Actual reimbursement expenditure	Reimbursement ratio
Policy	-0.006 (0.006)	-0.004 (0.006)	−0.041*** (0.007)	− 0.026*** (0.001)
Covariates variables	Y	Y	Y	Y
Time fixed effects	Y	Y	Y	Y
Hospital fixed effects	Y	Y	Y	Y
Observations	514,631	514,631	514,631	514,631
R-squared	0.21	0.25	0.26	0.26

Covariates variables include the gender, age, outcome, type of diseases, and so on. Robust standard errors are reported in parentheses, ***denotes the significance at the 1% level

At the same time, we found the results were significantly different from F,G. S. et al. [12]. They used the aggregated data provided by 12 hospitals in Beijing for analysis. The study found that the SHRDS policy significantly reduced the personal economic burden of patients by 23%. Why do the studies have different conclusions? On the one hand, the hospital data includes patients who do not have healthcare insurance. On the other hand, it also reflects that the summary data provided by the hospital may have serious measurement errors. Obviously, because the hospital is a direct stakeholder in the SHRDS policy, the summary data provided by the hospital is more likely to have measurement errors.

### Empirical results of the components of healthcare expenditure

As this study only examined the expenditure of drugs, examinations, supplies and materials as well as nursing, all of which account for a relatively high proportion in total healthcare expenditure. We can find that after the SHRDS policy, the drug expenditure has decreased significantly by 14.4% (e^− 0.144^-1, *p*-value = 0.000), indicating that the SHRDS policy has a significant effect on reducing the drug expenditure, which is conducive to reducing the economic burden of patients in medicines (Table 7). However, we also found that after the SHRDS policy, the examinations expenditure increased significantly by 18.2%(e^0.167^–1,*p*-value = 0.000), the materials expenditure increased significantly by 38.5%(e^0.326^–1,p-value = 0.000), and the nursing expenditure increased significantly by 12.7% (e^0.120^–1, p-value = 0.000), indicating that the SHRDS policy significantly increased the examinations and materials expenditure for patients. The possible reasons are: on the one hand, although the government implements SHRDS policy, it did not cancel the profit margin for materials sales at public hospitals, thus the hospitals make up for the loss from drug sales by increasing the sales of materials; on the other hand, in order to make up for the loss caused by the SHRDS policy, hospitals will increase the price of other healthcare services such as examinations and nursing to make profits, resulting in a corresponding increase in healthcare expenditure. At the same time, doctors induce patients’ healthcare needs and increase patients’ use of healthcare services to ensure the revenue of the hospital. It should be noted that the increase in the price of healthcare services can better reflect the labor value of healthcare staffs. If the increase in examinations and nursing expenditure is due to the government’s adjustment of the price of healthcare services to compensate for the SHRDS policy, this is the incentives of policy. The increase in healthcare expenditure brought about by the incentives of policy requires the government to balance the relationship between the income of hospitals and the economic burden of patients. The doctor-induced medical services may not be actually needed by patients; thus, it is an excessive demand induced by the supplies of hospitals. It requires the government to strengthen supervision of hospitals, improve the compensation mechanism after the SHRDS policy, and alleviate the patients’ healthcare economic burden. The SHRDS policy undoubtedly incentivized physicians to over-prescribe expensive drugs and tests, even when they were not clinically warranted [13].

**Table 7:** The structures of healthcare expenditure

Variables	Drug expenditure	Inspection expenditure	Material expenditure	Nursing expenditure
Policy	-0.144*** (0.009)	0.167*** (0.007)	0.326*** (0.012)	0.120*** (0.006)
Covariates variables	Y	Y	Y	Y
Time fixed effects	Y	Y	Y	Y
Hospital fixed effects	Y	Y	Y	Y
Observations	514,631	514,631	514,631	514,631
R-squared	0.16	0.28	0.04	0.05

Covariates variables include the gender, age, outcome, type of diseases, and so on. Robust standard errors are reported in parentheses, ***denotes the significance at the 1% level

**Fig. 7:**
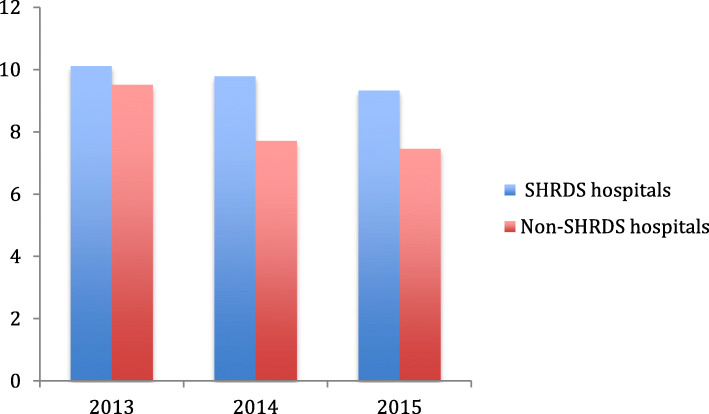
The length of hospital stay for patients in SHRDS hospitals and Non- SHRDS hospitals. Notes: The Y-axis shows the average length of hospital stay of the patients (unit: Days) and X-axis shows the year

**Fig. 8:**
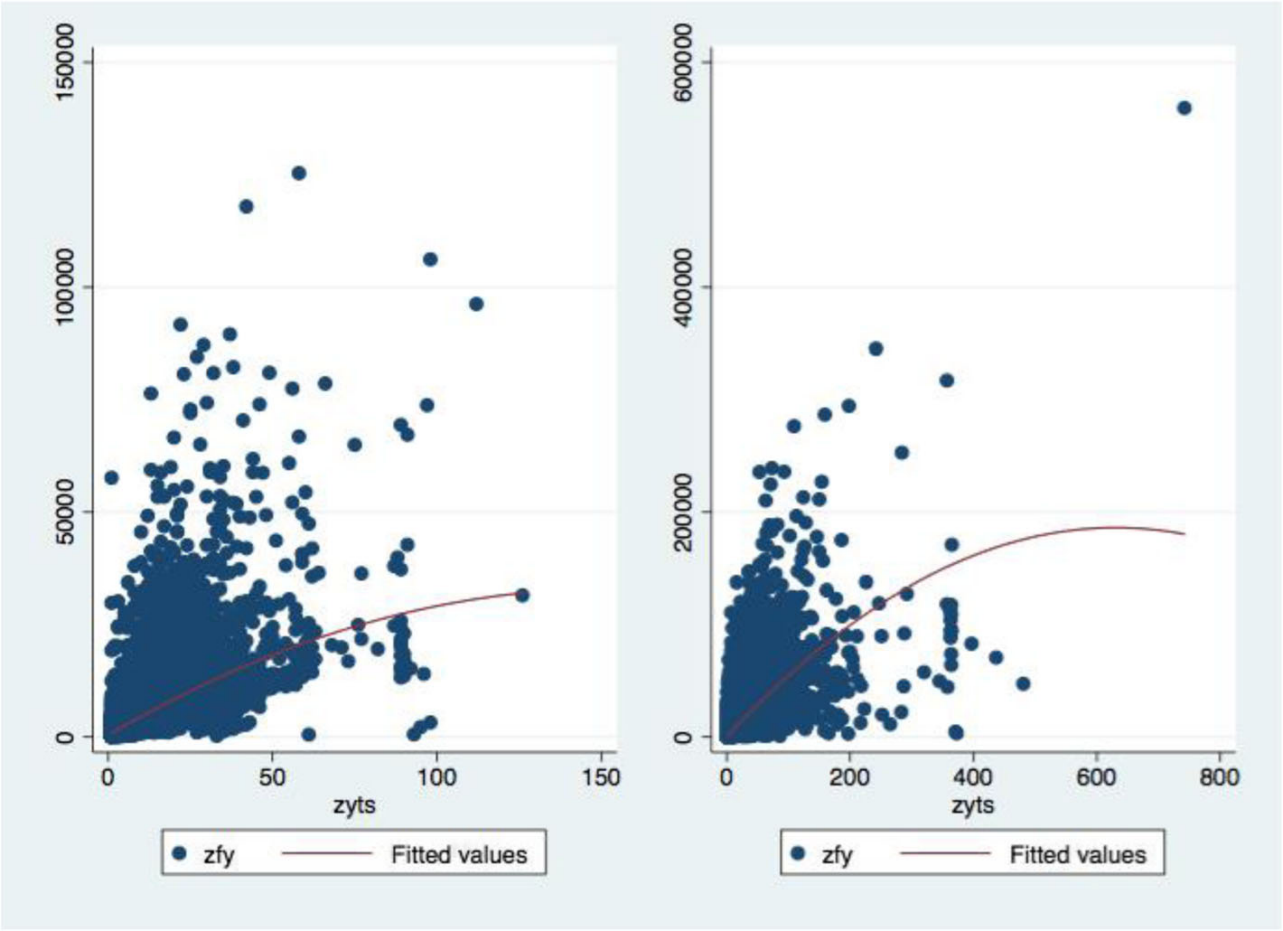
The scatter plot and quadratic regression line of total healthcare expenditures and average length of stay. Notes: The left side represents Non-SHRDS hospitals, the right side represents SHRDS hospitals, zfy denotes total healthcare expenditures (unit: Yuan [CNY]), and zyts denotes average length of stay (unit: Days)

The SHRDS policy has led to changes in the price of medicines and healthcare services, and the income of hospitals. In the case of the government adjusting the price of healthcare services as a compensation measure, whether the hospital (physicians) obtains excessive profits in order to maintain or increase healthcare income will induce excessive healthcare consumption by the patients. Although we are unable to directly observe the number of services that patients receive for inspection and nursing, we can observe the final healthcare expenditure. In order to answer this question, we can test the impact of the average length of hospital stay on the total healthcare expenditure. The underlying logic behind it is that healthcare examinations and nursing are sufficient to fully reflect the value of healthcare services for hospitals’ staffs [10]. In general, the greater the average length of hospital stay for patients, the more services and productures the consumes at the hospital; the more healthcare services such as examinations and nursing, the higher the total healthcare expenditure of the patient. If the SHRDS hospitals and the Non-SHRDS hospitals are in the same policy environment after the implementation of the SHRDS policy, the average length of stay of the SHRDS hospitals will increase significantly compared with the average length of stay of the Non-SHRDS hospitals, resulting in increased healthcare services such as examinations and nursing for patients. The increased healthcare services in quantity also validates our previous conjecture. From Fig. 7, we can find that the average length of stay of SHRDS hospitals are significantly higher than those of Non-SHRDS hospitals, especially in 2014 with the impact of the SHRDS policy. To further illustrate this problem, we have made a quadratic regression scatter plot (Fig. 8) for the total healthcare expenditure and average length of stay for SHRDS and Non-SHRDS hospitals. We can clearly see that the average daily increase of patients in the SHRDS hospitals is far greater than that of Non-SHRDS hospitals, the quadratic regression line, and the gap is still widening. Therefore, we have reasons to believe that there are hospitals (physicians) to induce patients’ healthcare needs, increase the use of healthcare services to ensure the hospitals’ income, and directly lead to an increase in the social healthcare economic burden. In particular, it should be noted that the increased social healthcare economic burden is ultimately passed on to the patients’ personal burden through the decline in the reimbursement ratio. In the practice of the SHRDS policy, the government must strengthen the effect of policy incentives on controlling healthcare expenditure and eliminating the drawbacks of hospital revenue from drug sales, and also need to achieve a balance between hospitals’ income and patients’ burden to ensure the SHRDS policy in the legitimate interests of the stakeholders.

## Discussion

### Main findings

The essence of the SHRDS policy is to gradually promote zero makeup drug policy, compensate the hospital revenue from government finance, implement the two-line management of the drugs revenue and expenditure, and cut off the economic connection between the hospital and the revenue from drug sales [14] (Gao,2005). In order to establish a medical and health system with Chinese characteristics, realize the goal of sharing basic healthcare services for all, and improve the health of the whole people, the government pointed out that we should promote the SHRDS policy, explore various effective ways to gradually abolish hospital revenue from drug sales, and change the compensation mechanism of public hospitals. Meanwhile, the government would increase investment for public hospitals, improve the economic compensation policy of public hospitals, and solve the problem of hospital revenue from drug sales.

In this study, we use the large sample data of urban employee’s healthcare insurance in Chengdu to evaluate the effect of the SHRDS policy by using the DID model, and estimate the impact of the SHRDS policy on healthcare expenditure and the economic burden for patients. We provide empirical evidence for deepening the medical and health system reform in China. This study had a number of important findings.

Firstly, in terms of total healthcare expenditure and reimbursement ratios, after the SHRDS policy, the total healthcare expenditure of patients decreased by only 0.6% (statistically significant at the 1% level), and the xeffect of the SHRDS policy on the total healthcare expenditure was very limited. The actual reimbursement expenditure of patients decreased significantly by 4.1%, and the reimbursement ratio decreased significantly by 2.6%. The SHRDS policy increased the healthcare economic burden of patients. Due to the problem of information asymmetry between physicians and patients, physicians can increase the demand for healthcare services through information superiority, thus increase income levels [15]. Li and Yu [16] found that it is difficult for patients to grasp comprehensive healthcare service information, and physicians are professionals, who essentially have monopoly power over healthcare information and resources, thus induce the demand of healthcare services in pursuit of personal interests. At the command of doctors, patients undergo excessive healthcare consumption. The phenomenon of supply-induced demand is also prevalent in USA, which is mainly manifested in the overuse of surgical services and medical examinations [17–20]. McGuire and Pauly [21] found that if physicians have a target income level, physicians have an incentive to increase the use of healthcare services by inducing the demand of patients to achieve a specific income level in the case that the price of healthcare services is regulated. The Chinese government imposed strict price controls on healthcare services. In order to increase their income, hospitals have strong economic incentives for over-prescription and over-treatment. Due to the existence of a 15% profit margin for drug sales, physicians induce patients to overuse drugs to become the main means for public hospitals to increase their income level [10]. Excessive healthcare expenditure has become a concentrated expression of the problem of health care’s prohibitive costs (commonly known as *kan-bing-gui* in Chinese).

Secondly, in terms of the structure of healthcare expenditure, after the SHRDS policy, the drug expenditure dropped significantly by 14.4%. The SHRDS policy has a significant effect on reducing the drug expenditure, which is, ceteris paribus, beneficial to alleviating the economic burden of patients in medicines. However, the examinations expenditure increased significantly by 18.2%, material expenditure increased significantly by 38.5%, and nursing expenditure increased significantly by 12.7%. The SHRDS policy significantly increased the expenditure of examinations, materials and nursing for patients. Although the SHRDS policy has reduced of the total healthcare expenditure and drug expenditure, it has increased the economic burden of examinations, materials and nursing expenditure for patients. Further tests found that there are hospitals (physicians) to induce the patients’ healthcare needs, increase the patients’ use of healthcare services to ensure the hospitals’ income. Duan and Liu [22] used the fuzzy comprehensive evaluation method to implement the Policy of SHRDS in Beijing. The patients were evaluated along four dimensions: healthcare quality, healthcare expenditure, healthcare time and healthcare convenience. Satisfaction with the healthcare quality and healthcare expenditure is high, but the SHRDS policy has formed a stagnation of drug price, which has reduced the satisfaction of patients’ healthcare time and healthcare convenience. Wang and Du [23] used the doctors’ and hospitals’ income as a mediator to evaluate the impact mechanism of the SHRDS policy on healthcare expenditure. It was found that the SHRDS policy indirectly affected the income of hospitals and physicians, and indirectly affected the drug expenditure and efficiency of resources allocation. Compared with the financial subsidies for healthcare, the effect of adjusting the price of healthcare services on income is more significant. The increase in hospitals’ and physicians’ income will drive further healthcare expenditure. Subsidizing hospitals’ income can delay the upward trend in healthcare expenditure compared with subsidized physicians’ income. Wang and Nan [24] analyzed the impact of the SHRDS policy on the supply of healthcare services by constructing a model of intermediary variables. The study found that optimizing the supply of healthcare services is urgently needed to return to the essence of healthcare services. Zhang et al. [25] estimated the impact of the SHRDS policy on the hospitalization expenditure of rural patients. The study found that compared with the inpatients in Non-SHRDS counties, the total healthcare expenditure of inpatients in SHRDS counties increased significantly by 28.7%. Drug expenditure dropped significantly by 9.5%, but examinations and treatment expenditure have increased significantly. Zhuang et al. [26] evaluated the impact of Beijing’s SHRDS policy on the total healthcare expenditure of medical institutions. The study found that the SHRDS policy effectively controlled the excessive growth of emergency services. Zhou et al. [27] used intermittent time series analysis to evaluate the impact of Beijing’s SHRDS policy on the medical treatment of emergency patients. The study found that the average number of emergency department visits per hospital in the tertiary hospitals decreased by 12.11%.

Finally, after the SHRDS policy, the significant reduction in drug expenditure led to more physicians inducing patients’ healthcare service needs. These findings are consistent with the theory of physicians agency [28, 29]. Simultaneously, We had the most important finding which is different from other researches. The SHRDS policy increased social healthcare burden and it was partially transferred to the patients’ personal burden through the decline in the reimbursement ratios. Wang et al. [30] used ischemic heart disease as an example to evaluate the impact of Beijing’s SHRDS policy on physicians’ treatment behavior and healthcare expenditure in five SHRDS hospitals. The study found that the SHRDS policy changed the structure of expenditure in hospitals, reduced the total healthcare expenditure, and other expenditure such as drug expenditure, and realized the shift of income. Under the existing healthcare insurance payment and hospital income distribution system, the SHRDS policy has little effect on the total healthcare expenditure and the patient’s self-pay. Strengthening the internal management of hospitals and strictly controlling performance indicators have a certain effect on regulating physicians’ behavior. Xie et al. [31] used public hospitals of Beijing as an example to analyze the influencing factors of healthcare expenditure. The study found that patients’ burden of healthcare expenditure remained balanced before and after the SHRDS policy. There was no significant difference in the healthcare expenditure between self-paying patients and healthcare insurance patients. Chen et al. [10] based on healthcare insurance reimbursement data, estimated the impact of the SHRDS policy on healthcare expenditure levels and expenditure structure. The study found that the SHRDS policy significantly increased the total healthcare expenditure of patients by 4.9%, resulting in a significant decrease in patient drug expenditure by 9.5%, and a significant increase in nursing and treatment expenditure by 69.7 and 53.4%. The SHRDS policy in public hospital has improved the income structure of public hospitals, but it has not reduced the healthcare expenditure, nor has it significantly reduced the burden of healthcare expenditure for patients.

In summary, the current research conclusions on the effect of China’s SHRDS policy are not uniform, mainly for the following reasons:(1) The data source is mainly the summary data provided by the hospital. Due to the hospital as a stakeholder in the SHRDS policy, the data provided may have problems such as measurement error and misrepresentation; (2) The research city is mainly in Beijing, and there are few studies on the of SHRDS policy in other regions or cities. As a major national policy of medical and health system reform, the SHRDS policy is of great significance for the adjustment and improvement of the SHRDS policy for other regions and cities; (3)The research method mainly adopts comparative analysis of data before and after the SHRDS policy, lacking scientific and effective measurement and analysis methods reduce the reliability and effectiveness of research results and conclusions; (4)The number of data samples used in the study is small, and the use of big data to evaluate the effect of the SHRDS policy is not evaluated.

### Strengths

This paper evaluates the effect of the SHRDS policy. There are four main contributions:(1) The data is based on the healthcare insurance settlement data provided by Healthcare Insurance Administration of Chengdu, which can ensure the authenticity of the data and avoid measurement errors and false reports; (2) Using data from the personal settlement of urban employees’ healthcare insurance in Chengdu, as a research sample, providing empirical evidence for other cities to deepen the reform of China’s medical and health system; (3) Adopting a difference in difference model (DID) for the SHRDS policy. The effects of the SHRDS policy are evaluated, and a variety of methods are used for causality testing. The findings and conclusions are more reliable and effective; (4) Using the large sample data of urban employee’s healthcare insurance settlement in Chengdu, the individual characteristics can be better controlled, the analysis is more rigorous, and the conclusion is more reliable.

### Policy implications

All of these findings imply that the SHRDS policy is not an effective way to control healthcare expenditure. However, while the government is pursuing the SHRDS policy, it is necessary to improve the system of financial subsidies and adjust the price of healthcare services, balance the relationship between hospitals’ income and patients’ burden, and avoid physicians inducing patients to accept excessive healthcare service demand. This is the key issue that needs some attention in the future reform of public hospitals.

On the contrary, the SHRDS policy increased economic healthcare burden for patients. When considering the SHRDS policy, policymakers may also need to monitor the interests for hospitals and patients that may be unintentionally impacted. When the government implements the SHRDS policy, policymakers have to pay attention to the synergy among different policies. The government should strengthen public hospitals reform, healthcare integrated system, and healthcare payment methods to build a coordinated management structure, price system, and incentive mechanism. Building a balanced benefit-centric integrated service supply model for people will be better meet the changing medical service needs and achieve the goal of providing high quality and sustainable medical services in healthcare system reform.

## Conclusion

In general, the SHRDS policy has a significant impact on the structure of healthcare expenditure. The government compensates the hospitals’ losses by making financial subsidies and adjusting the price of health services. The SHRDS policy has no significant effect on controlling or reducing healthcare expenditure, but it increases the personal economic burden of patients. Because the data in this study can’t directly observe the quantity and quality of healthcare services received by patients, it can’t test the supply-induced excessive healthcare needs in the healthcare process. This is also an important direction for some research of the SHRDS policy in the future.

## Data Availability

Data and materials are from Healthcare Security Administration of Chengdu City.

## Abbreviations

SHRDS: Separating of hospital revenue from drug sales
HIR: Healthcare insurance reimbursement
DID: Difference-in difference model

## References

[1] Fan VY, Savedoff WD (2014). The health financing transition: a conceptual framework and empirical evidence. Soc Sci Med.

[2] Yip WC, Hsiao WC, Chen W, Hu S, Ma J, Maynard A (2012). Early appraisal of China’s huge and complex health-care reform. Lancet.

[3] Seiter A, Wang H, Zhang S (2010). A Generic Drug Policy as a Cornerstone to Essential Medicines in China. The World Bank Research Report.

[4] Currie J, Lin W, Meng J (2014). Addressing Antibiotic Abuse in China: An Experimental Audit Study. J Dev Econ.

[5] Yang L, Ying C, Sufang G, Brant P, Bin L, Hipgrave D (2013). Evaluation,in Three Provinces, of the Introduction and Impact of China’s National Essential Medicines Scheme. Bull World Health Organ.

[6] Yip W, Hsiao WC (2008). The Chinese health system at a crossroads. Health Affairs.

[7] David C, B Krueger A (1994). Minimum Wages and Employment: A Case Study of the Fast-food Industry in New Jersey and Pennsylvania. Am Econ Rev.

[8] Esther D (2001). Schooling and labor market consequences of school construction in indonesia: evidence from an unusual policy experiment. Am Econ Rev.

[9] Zhang C, Giles J, Zhao Y (2014). Policy evaluation of China’s new rural pension program: income, poverty, expenditure, subjective wellbeing and labor supply. China Econ Quart.

[10] Chen Z, Song Z, Zhang CC (2018). Effect of separating treatment and drug sales: evidence form medical insurance claims data. J Fin Res.

[11] Zhang Y, Ma Q, Chen Y, Gao H (2017). Effects of Public Hospital Reform On Inpatient Expenditures in Rural China. Health Econ.

[12] F GS, Zhu HP, F MW. An empirical analysis of the effect of the pharmaceutical separation reform. Chinese Journal of Hospital Administration. 2014;12:881-5.

[13] Yip W, Hsiao W (2008). China’s health care reform: a tentative assessment. China Econ Rev.

[14] Gao Q. Comprehensively establish and implement the scientific development concept, and promote the reform and development of the health industry—The work report in national health work conference at 2005. Beijing: Ministry of Health; 2005.

[15] Pauly MV (1968). The Economics of Moral Hazard: Comment. Am Econ Rev.

[16] Li LL, Yu Q (2019). The impact of the reform of the basic medical insurance payment method on medical expenses in China. Comp Econ Soc Syst.

[17] Farley PJ (1986). Theories of the Price and Quantity of Physician Services: A Synthesis and Critique. J Health Econ.

[18] Rice TH, Labella RJ (1989). Do physicians induce demand for medical services?. J Health Polit Policy Law.

[19] Yip WC (1998). Physician Response to Medicare Fee Reductions: Changes in the Volume of Coronary Artery Bypass Graft (Cabs) Surgeries in the Medicare and Private Sectors. J Health Econ.

[20] Clemens J, Gottlieb JD (2014). Do physicians’ financial incentives affect medical treatment and patient health?. Am Econ Rev.

[21] McGuire TG, Pauly MV (1991). Physician Response to Free Changes with Multiple Payers. J Health Econ.

[22] Duan H, Liu C (2015). Patients’ satisfaction with the medical services under the medicine and medical services separation policy: a fuzzy comprehensive evaluation. Pub Admin Policy Rev.

[23] Zhang CC, Lei XY, John S, Zhao YH (2017). Health Insurance and Health Care Among the Mid-aged and Older Chinese: Evidence from the National Baseline Survey of CHARLS. Health Econ.

[24] Wang WJ, Du JJ (2015). The influence mechanism of separation of clinic from pharmacy on medical expense—mediated effects of doctor income and hospital income. Chin Soft Sci.

[25] Wang WJ, Nan MX (2016). Return to the nature of medical services: an analysis of medical services supply from the perspective of medical separation. Modern Econ Sci.

[26] Zhuang Y, Zhou SD, Yang S (2017). The impact of separating drug sales from medical services on mechanism of controlling outpatient and emergency expenses in Beijing. Chin J Health Policy.

[27] Zhou SD, Zhuang Y, Yang S (2018). The comprehensive reform of separating drug sales from medical services and its impact on outpatients and emergency medical flow in Beijing. Chin J Health Policy.

[28] Arrow KJ (1963). Uncertainty and the welfare economics of medical care. Am Econ Rev.

[29] McGuire TG (2000). Physician agency. Handbook Health Econ.

[30] Wang JF, Chen ZC, Cui B (2018). The impacts of separating drug sales from medical services pilots on inpatient expenses in Beijing: A case study of five pilot tertiary hospitals. Chin J Health Policy.

[31] Xie ST, Cao G, Shen H (2019). Study on the evaluation of cost control effect in public hospital during the separation of clinic from pharmacy reform. Chin Hosp Manag.

